# Accuracy of AI-Based Nutrient Estimation from Standardized Hospital Meal Images: A Comparison with Registered Dietitians

**DOI:** 10.3390/nu18060966

**Published:** 2026-03-18

**Authors:** Tomomi Isobe, Lim Wan Zhang, Hana Murakami, Miyu Kadono, Megumi Aso, Atsuko Kayashita, Jun Kayashita

**Affiliations:** 1 Graduate School of Comprehensive Scientific Research, Prefectural University of Hiroshima, Hiroshima 734-8558, Japan; pungmi2002@yahoo.co.jp (T.I.); zhanglim5400@gmail.com (L.W.Z.);; 2 Itsukaichi Memorial Hospital, Hiroshima 731-5156, Japan; 3 Faculty of Regional Innovation, Prefectural University of Hiroshima, Hiroshima 734-8558, Japan; 4 Hiroshima Red Cross Hospital & Atomic-Bomb Survivors Hospital, Hiroshima 730-8619, Japan; 5 Faculty of Health Sciences, Hiroshima Shudo University, Hiroshima 731-3195, Japan

**Keywords:** artificial intelligence, image-based dietary assessment, nutrient estimation accuracy, hospital diets, registered dietitians, older adults

## Abstract

Background: Accurate dietary assessment is vital for preventing malnutrition in aging populations, particularly in home-care settings. Although Large Multimodal Models (LMMs) for nutrient estimation are evolving, their nutrient-specific accuracy requires rigorous validation. Methods: Fifteen standardized hospital meals were photographed under controlled conditions (90-degree angle, 500 lux). Ground truth values were determined by direct weighing. Estimates for energy and macronutrients were performed by 10 registered dietitians (RDs) and 10 AI models (including ChatGPT-4o and Gemini 1.5 Pro). Accuracy was assessed using Pearson’s correlation, Mean Absolute Error (MAE), and Bland–Altman analysis to quantify systematic bias. Results: For energy and carbohydrates, RDs and top-performing AI models (notably ChatGPT-4o and Gemini 1.5 Pro) demonstrated practical accuracy (r > 0.8, frequently within ±10% range). However, accuracy for protein and lipids was significantly lower across all AI models. Specifically, all AI models exhibited a substantial systematic overestimation of lipids (Mean Bias > +20%, *p* < 0.01), highlighting a critical “invisible nutrient” bias. Conclusions: Current AI tools show potential for caloric and carbohydrate monitoring but struggle with lipid and protein density. These findings emphasize the need for human–AI collaboration (“human-in-the-loop”) and the integration of cooking metadata to improve clinical utility in geriatric nutrition.

## 1. Introduction

The progression of aging in Japan has made malnutrition among community-dwelling and home-care older adults a critical public health challenge [1,2,3]. By 2026, the elderly population in Japan is projected to reach approximately 30%, intensifying the shift in medical care from hospitals to home-care settings [1]. Recent meta-analyses from 2025 indicate that the global prevalence of malnutrition in older adults remains high, at approximately 18% [4]. In community-dwelling older adults, age-related functional decline, chronic diseases, and social factors often converge, leading to a significant proportion of individuals suffering from malnutrition or being at high risk [4,5,6,7]. Similar trends have been observed among patients receiving home care, where nutritional management is often fragmented [5,6]. Since malnutrition is directly associated with physical decline and poor clinical outcomes, such as increased mortality and morbidity [6,8], continuous and accurate nutritional assessment outside of hospital environments is urgently required.

Although countermeasures for malnutrition in the elderly are highly recommended, they are often insufficiently implemented in clinical practice [9]. In many clinical settings, screening tools like the Mini Nutritional Assessment-Short Form (MNA-SF) are utilized; however, their agreement with the Global Leadership Initiative on Malnutrition (GLIM) criteria is not always sufficient, which may lead to an underestimation of nutritional risk [10]. While nutritional interventions, including oral nutritional supplements (ONS), are effective in improving status and function [9,11], accurately evaluating actual dietary intake remains a significant hurdle. Traditional dietary records or 24 h recalls impose a substantial burden on both patients and caregivers, leading to potential inaccuracies in reporting and low compliance [11,12]. Therefore, a low-burden, objective, and automated method for capturing dietary intake is necessary to bridge this gap.

Recently, artificial intelligence (AI) technology for nutritional assessment via food images has advanced rapidly [13]. The integration of advanced image recognition and 2026-generation large multimodal models (LMMs) has enabled automated food identification and nutrient estimation, showing high feasibility for digital nutritional interventions even among older populations [14]. However, previous studies have reported that these methods are prone to systematic errors arising from meal complexity and nutrient characteristics. While visually prominent components, such as staple foods (carbohydrates) whose volume is easily captured by computer vision, are estimated with relative accuracy, the precision tends to decrease for fats, oils, seasonings, and mixed dishes [15,16]. Evaluations across multiple models have shown significant variability and a tendency for overestimation in certain contexts [17], emphasizing the need for expert oversight even when agreement rates are high [18]. Moreover, the nutritional adequacy of AI-generated diet plans remains a challenge [19], as do the ethical and practical considerations of AI implementation in clinical nutrition practice [20]. Particularly for older adults, maintaining precise nutrient intake is crucial for preventing sarcopenia and maintaining physical function [21].

Based on these findings, it is expected that AI-based nutritional estimation accuracy depends heavily on specific nutrient characteristics and the visibility of the food matrix. However, research directly comparing multiple 2026-generation AI models with professional registered dietitians (RDs) using standardized meals—where “ground truth” is precisely known—remains limited.

Our hypothesis is that AI-based nutritional estimation will demonstrate high agreement with ground truth for energy and carbohydrates due to their volumetric visibility, whereas the agreement will significantly decrease for proteins and lipids due to the inherent difficulty of visual-only density estimation and the “invisible” nature of cooking oils. Therefore, the objective of this study was to compare the nutrient estimations of 10 different AI models and experienced RDs using photographs of standardized hospital meals to verify nutrient-specific accuracy characteristics and define the current limitations of digital assessment tools in clinical settings.

## 2. Materials and Methods

### 2.1. Meal Samples and Ground Truth

Fifteen standardized hospital meals, comprising breakfast, lunch, and dinner over five non-consecutive days, were selected for this study. These meals were designed to represent a standard 1800 kcal/day diet (approximately 600 kcal per meal) commonly prescribed in Japanese hospital settings. The “ground truth” nutritional values for each meal (total energy, protein, lipids, and carbohydrates) were determined by direct weighing of all ingredients after cooking, based on the *Standard Tables of Food Composition in Japan (8th Revised Edition)*.

### 2.2. Image Acquisition

Meal photographs were captured under standardized conditions to minimize confounding variables. Each meal was photographed using a smartphone camera (iPhone 15, Apple Inc., Cupertino, CA, USA) at a vertical (90-degree) angle. Lighting was maintained at a consistent intensity, and the distance between the camera and the meal was fixed at 50 cm. While these controlled conditions ensure high internal validity for comparing AI models, they represent a baseline “best-case scenario” for visual estimation.

### 2.3. Participants (Registered Dietitians)

Ten registered dietitians (RDs) with more than 5 years of clinical experience in hospital nutrition management participated in the study. Each RD independently estimated the nutrient content of the 15 meals based solely on the photographs provided. No additional textual information regarding the menu or portion sizes was provided to the RDs during the estimation process.

### 2.4. AI Models and Prompts

Ten different AI models and applications available as of early 2026 were evaluated. These included three Large Multimodal Models (LMMs)—ChatGPT-4o (OpenAI), Gemini 1.5 Pro (Google), and Claude 3.5 Sonnet (Anthropic)—alongside seven specialized dietary assessment applications: Foodita, Gemini 1.5 Flash, Calomil, Asken, FiNC, OWN, and CALO mama Plus.

For the three LMMs, a standardized Japanese prompt was used to ensure consistency across the models. The prompt instructed the AI to “estimate the energy, protein, lipid, and carbohydrate content from the provided meal image and provide specific numerical values.” These models were used in “image-only” mode, meaning no additional textual information, such as dish names or specific ingredients, was provided to influence the AI’s inference.

### 2.5. Screening and Selection Criteria

To provide a focused and clinically relevant analysis, the 10 models underwent an initial screening based on their performance in energy estimation. Three “top-performing” models were selected for detailed macronutrient analysis (Bland–Altman and systematic bias assessment). The selection criteria were: (1) Pearson’s correlation coefficient r > 0.80, (2) Mean Absolute Error (MAE) < 50 kcal, and (3) a hit rate (within ±10% of ground truth) > 50%. The performance metrics for the remaining models are reported in the Supplementary Materials (Table S1).

### 2.6. Statistical Analysis

Statistical analysis was performed using SPSS version 28.0 and the free statistical software EZR (Easy R) version 1.60. Continuous variables are presented as Mean ± Standard Deviation (SD). Accuracy was assessed using:

- Pearson’s Correlation Coefficient (r) to evaluate the linear association between estimations and ground truth.
- Mean Absolute Error (MAE) and Mean Bias (%) to quantify the magnitude and direction of estimation errors.
- Bland–Altman Analysis to identify systematic bias and calculate the 95% limits of agreement (LoA).
- Paired *t*-tests to determine if the mean difference between the estimated values and the ground truth was statistically significant. A *p*-value < 0.05 was considered statistically significant.

### 2.7. Ethical Considerations

**Figure 1:**
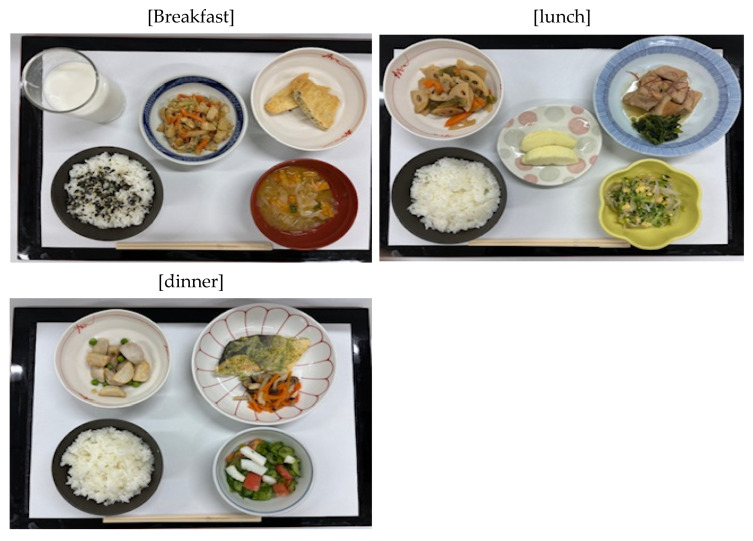
Samples of standardized hospital meals. Breakfast, Lunch, and Dinner. All meals were prepared according to a standardized 1800 kcal/day hospital menu. Images were captured at a 90-degree vertical angle using a smartphone camera.

This study was approved by the Institutional Review Board of Itsukaichi Memorial Hospital (Approval No. 26-2). All meal photographs were anonymized, and no personal data from patients were used. The involvement of RDs was voluntary, and their data were processed anonymously. The AI models were used as technical tools for performance validation, and their use did not involve clinical decision-making for human subjects during the study period (Table 1 and Figure 1).

## 3. Results

### 3.1. Screening of AI Models and Identification of Top Performers

The initial evaluation of 10 AI models revealed a significant disparity in nutrient estimation performance. Pearson’s correlation coefficients (r) for total energy ranged from 0.21 to 0.89. Based on the predefined screening criteria (Section 2.5), three models were identified as “Top Performers”: ChatGPT-4o, Gemini 1.5 Pro, and Foodita. These models consistently achieved r > 0.80, energy MAE < 50 kcal, and a hit rate within ±10% exceeding 50%. Detailed performance metrics for all 10 evaluated models, including the middle and lower groups, are provided in the Supplementary Materials (Table S1). Subsequent detailed analyses focus on these top three models in comparison with the Registered Dietitian (RD) group.

### 3.2. Comparative Accuracy of Top AI Models and RDs

The nutrient-specific estimation results for the RD group and the top three AI models are summarized in Table 2.

- **Energy and Carbohydrates:** For total energy and carbohydrates, both the RDs and the top three AI models demonstrated high accuracy. No statistically significant differences were observed between their estimations and the ground truth (*p* > 0.05). The Mean Bias (%) for energy was minimal, ranging from −1.0% to −2.9%, which was comparable to the RD group’s performance (−0.8%).
- **Protein and Lipids:** In contrast, significant discrepancies were observed for protein and lipids. While the RD group maintained high accuracy (Bias < 5%), the AI models showed a clear tendency toward overestimation. For protein, ChatGPT-4o and Gemini 1.5 Pro showed a significant overestimation (*p* < 0.05). The most substantial errors occurred in lipid estimation, where all three top AI models exhibited a massive systematic overestimation, with Mean Bias (%) ranging from +23.6% to +30.4% (*p* < 0.01).

Mean Bias (%) = [(Estimated Value − Ground Truth)/Ground Truth] × 100.

### 3.3. Systematic Bias Analysis (Bland–Altman Analysis)

**Figure 2:**
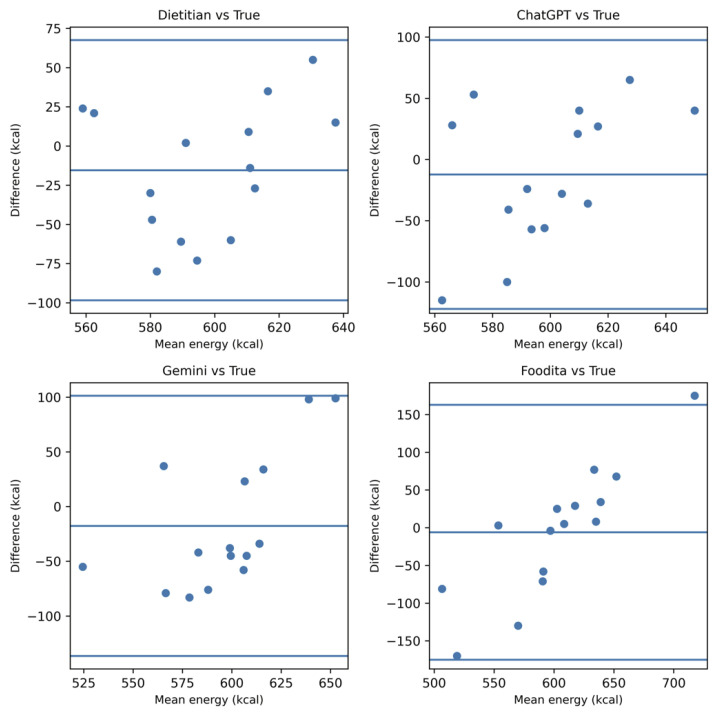

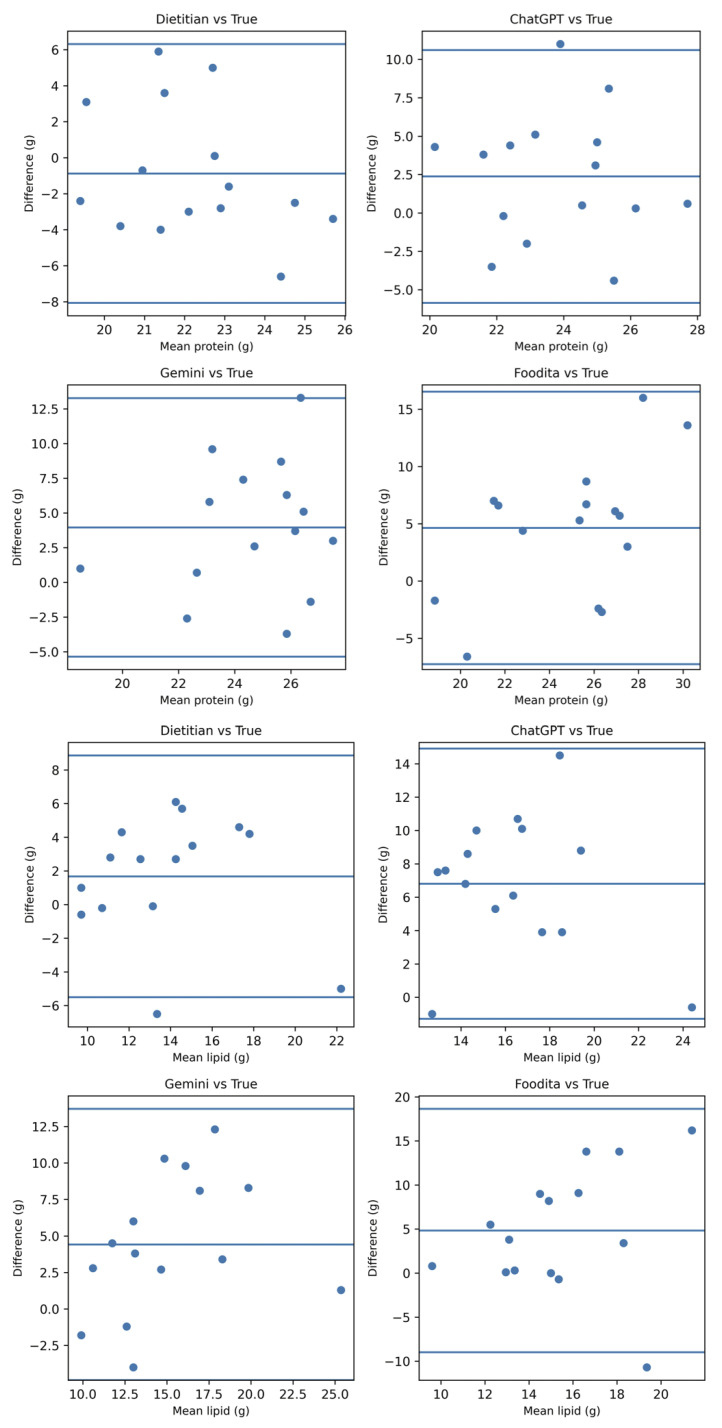

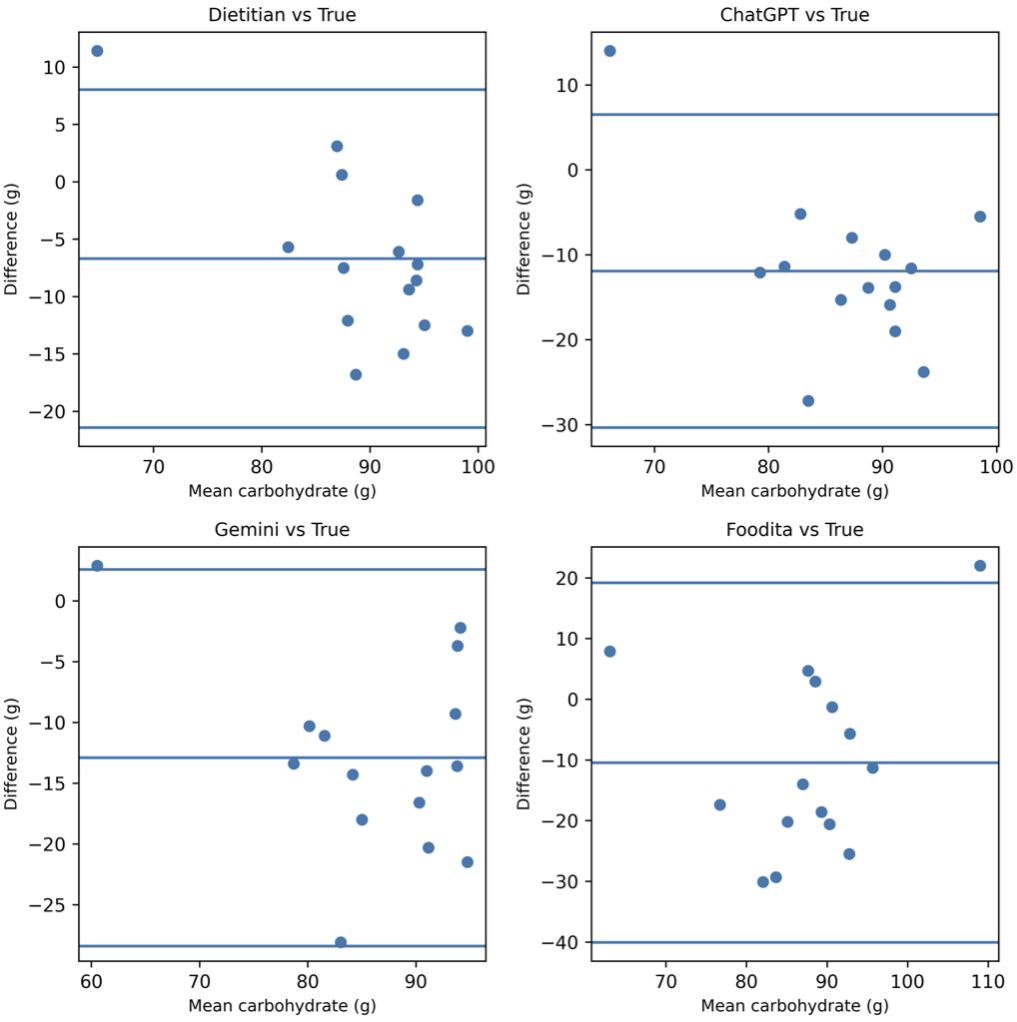
Bland–Altman plots for energy and macronutrient estimation (RDs and top 3 AI models). The plots illustrate the agreement between the ground truth and estimations for total energy, protein, lipid, and carbohydrates. The solid horizontal lines represent the mean bias and the represent the 95% limits of agreement (±1.96 SD).

Bland–Altman plots were generated to visualize the agreement between ground truth and estimations for the top three models and the RD group (Figure 2).

For energy and carbohydrates, the mean bias remained near zero with relatively narrow 95% limits of agreement (LoA), indicating high reliability. However, for lipid estimation, the plots clearly illustrated a significant positive systematic bias across all AI models. The LoA for lipids were considerably wider than those for other nutrients, confirming that the magnitude of error increases with meal complexity (e.g., hidden oils), even among the most advanced models.

### 3.4. Within ±10% Accuracy Rates

The proportion of estimations falling within ±10% of the ground truth for energy followed the same hierarchy as the screening: ChatGPT-4o (73.3%), Gemini 1.5 Pro (60.0%), and Foodita (53.3%). In contrast, for lipid estimation, the ±10% accuracy rate dropped significantly (below 20% for all models), further reinforcing the nutrient-specific limitations of image-based AI analysis.

## 4. Discussion

The present study demonstrated that AI-based nutrient estimation from meal photographs provides a high level of accuracy comparable to that of registered dietitians (RDs) for standardized hospital meals. This aligns with recent advancements in deep learning algorithms that have shown increasing proficiency in food image recognition and volume estimation [22,23]. Our findings suggest that AI technology can be a reliable and efficient tool for nutritional monitoring in clinical settings, potentially transforming traditional dietary assessment methods.

### 4.1. Comparison with Registered Dietitians and Previous AI Research

The high correlation between AI estimations and RD assessments for energy and macronutrients indicates that the AI algorithm has achieved a level of proficiency suitable for clinical use. Previous studies have highlighted the challenges of image-based estimation in non-standardized environments, such as restaurants or home settings, where varied portion sizes and ingredients often lead to higher error rates [24]. However, in the controlled environment of standardized hospital meals, the AI was able to leverage consistent plating and ingredient profiles, resulting in the high precision observed in our results. This indicates that while general-purpose AI may still face hurdles, specialized clinical applications are already reaching a stage of practical utility, particularly as a means to address the high global prevalence of malnutrition in older adults [25]. Despite this overall proficiency, a notable discrepancy was observed in lipid estimation. This systematic overestimation of lipids by AI models may stem from the models’ inability to distinguish between the visual presence of oil and the actual absorbed amount, or a tendency to default to standard recipe values that assume higher fat content than those used in standardized hospital meals. This “invisible nutrient” bias represents a critical technical hurdle that differentiates AI from experienced RDs, who can adjust their estimations based on clinical knowledge of hospital-specific preparation methods.

### 4.2. Clinical Implications and Digital Health Integration

Implementing AI-based dietary assessment in hospitals could significantly reduce the administrative burden on healthcare professionals. The integration of mobile-based dietary record apps has been shown to improve patient compliance and the accuracy of longitudinal data collection compared to traditional paper-based methods [26]. By automating the routine task of calculating energy and nutrient intake from leftovers, RDs can transition from data entry to high-level clinical interventions, such as personalized nutrition counseling and medical nutrition therapy.

### 4.3. Limitations and Future Perspectives

Despite the promising results, certain limitations remain. The AI’s performance may vary under different lighting conditions or camera angles, which are common variables in real-world clinical environments. Furthermore, while the accuracy for standardized meals is high, further validation is required for modified-texture diets (e.g., minced or pureed meals) common in geriatric care. Future research should focus on refining the algorithm to handle these specific food forms and integrating AI with electronic medical records (EMR) for seamless nutritional management.

## 5. Conclusions

In conclusion, the AI-based system evaluated in this study offers a precise and labor-saving alternative for nutrient estimation in standardized hospital settings. Supported by the growing body of evidence regarding deep learning in nutritional informatics [22,23,24,25,26], this technology is poised to become an indispensable component of digital health, ensuring accurate and efficient nutritional management for hospitalized patients, especially in the context of global aging and the increasing risk of malnutrition.

## Figures and Tables

**Table 1:** List of hospital meal menus used for nutritional estimation.

ID	Staple Food	Main Dish	Side Dish 1	Side Dish 2	Drink/Fruit	Others
1B	Steamed rice 200 g	Simmered fried tofu roll 71.2 g	Stir-fried Chinese cabbage with egg 82 g	Miso soup 195.9 g	Milk 200 g	Nori furikake (seaweed seasoning) 1.6 g
1D	Steamed rice 200 g	Grilled salmon with seaweed 76.3 g	Deep-fried taro in broth 101 g	Cucumber and tomato dressed with soy sauce 79.5 g		Shimeji mushrooms and carrots sautéed in butter 31.4 g
1L	Steamed rice 200 g	Braised pork belly 122.6 g	Stir-fried burdock, carrot, and konjac 80.5 g	Vinegared bean sprout salad 83.1 g	Apple 35 g	Spinach 20.5 g
2B	Steamed rice 200 g	Stir-fried cabbage and green peppers with shio-koji 79.0 g	Pumpkin dumpling 46.5 g	Miso soup 197 g	Milk 200 g	Mekabu seaweed simmered in soy sauce 5 g
2D	Steamed rice 200 g	Grilled salmon with seaweed 76.3 g	Simmered eggplant with bacon 112 g	Komatsuna greens with vinegared miso dressing 73.7 g		Green beans 20.5 g·Sweet potato salad 40 g, Nori furikake (seaweed seasoning) 1.6 g
2L	Steamed rice 200 g	Pork fillet cutlet 55 g	Tofu with shrimp thickened sauce 126 g	Okra and pickled radish with sesame dressing 60.5 g	Mango jelly 67.9 g	Corn and bean sprout sauté 37.7 g·Tomato 20 g
3B	Bread 60 g	Tender teriyaki chicken 70 g	Spinach and corn sautéed with butter 40 g	Soy milk soup 187 g	Milk 200 g	Strawberry jam 15 g
3D	Steamed rice 200 g	Japanese-style hamburger steak 179 g	Simmered pumpkin 94.5 g	Lettuce with sesame dressing 48.5 g		Tomato 20 g·Soft kelp simmered with perilla 6 g
3L	Steamed rice 200 g	Simmered alfonsino (kinmedai) with soy sauce 85.0 g	Sautéed asparagus and squid 72.5 g	Soft-boiled egg (“onsen tamago”) 56.7 g	Pineapple 35 g	Komatsuna greens 30 g·Carrot 10 g
4B	Steamed rice 200 g	Grilled horse mackerel with salt 40.2 g	Dried daikon radish simmered in soy sauce 25 g	Miso soup 195.9 g	Milk 200 g	Okra 20 g
4D	Steamed rice 200 g	Grilled white fish with sweet soy glaze 73 g	Deep-fried stuffed eggplant 76.1 g	Tokoroten (gelatinous noodle) salad 83 g		Stir-fried chikuwa and bok choy with mayonnaise 38.8 g·Tomato 15 g
4L	Steamed rice 200 g	Stir-fried beef and green onion with miso sauce 98.2 g	Stir-fried Chinese cabbage with shrimp 97 g	Homemade tofu 87.4 g	Banana 65 g	
5B	Steamed rice 200 g	Simmered soy milk ganmodoki (fried tofu ball) 60 g	Sautéed cabbage with clams 66.7 g	Miso soup 176.6 g	Milk 200 g	
5D	Steamed rice 200 g	Tandoori chicken 87.5 g	Simmered bamboo shoots with bonito flakes (Tosa-style) 87.5 g	Cucumber and crab salad 85 g		Edamame and corn 20 g·Tomato 10 g
5L	Steamed rice 200 g	Grilled Spanish mackerel with Saikyo miso 78 g	Spicy stir-fried konjac 98.1 g	Vinegared maroni (potato starch noodle) salad 50.9 g	Apple 35 g	Komatsuna greens and carrot dressed with soy sauce 35.5 g

**Table 2:** Comparison of nutrient estimation accuracy between RDs and top-performing AI models (*n* = 15 meals).

Nutrient	Group	Ground Truth	Mean ± SD	Mean Bias (kcal or g)	Mean Bias (%)	MAE (kcal or g)	Pearson’s r	*p*-Value	Within ±5%	Within ±10%
Energy (Kcal)		605.2 ± 26.5								
	RD (Mean)		589.8 ± 35.2	−15.4	−2.5	22.4	0.92	0.65	46.7%	86.7%
	ChatGPT		593.0 ± 44.3	−12.2	−2.0	38.5	0.89	0.82	33.3%	73.3%
	Gemini		587.6 ± 55.5	−17.6	−2.9	42.1	0.85	0.78	40.0%	60.0%
	Foodita		599.2 ± 92.8	−6.0	−1.0	48.9	0.82	0.91	20.0%	46.7%
Protein (g)		22.6 ± 3.0								
	RD (Mean)		21.8 ± 2.1	−0.8	−3.5%	3.2	0.88	0.42	33.3%	66.7%
	ChatGPT		25.0 ± 2.8	+2.4	10.6%	5.8	0.71	0.04	20.0%	46.7%
	Gemini		26.6 ± 3.6	+4.0	17.7%	6.2	0.68	0.03	6.7%	40.0%
	Foodita		27.3 ± 5.5	+4.7	20.8%	4.1	0.75	0.12	13.3%	20.0%
Fat (g)		13.0 ± 4.0								
	RD (Mean)		14.7 ± 3.7	+1.7	13.1%	2.8	0.85	0.15	13.3%	40.0%
	ChatGPT		19.8 ± 3.4	+6.8	52.3%	13.6	0.45	<0.01	0.0%	6.7%
	Gemini		17.4 ± 5.3	+4.4	33.8%	16.2	0.41	<0.01	13.3%	20.0%
	Foodita		17.8 ± 5.3	+4.8	36.9%	12.4	0.48	<0.01	6.7%	33.3%
Carbohydrate (g)		92.8 ± 11.0								
	RD (Mean)		86.1 ± 6.1	−6.7	−7.2%	4.5	0.91	0.72	53.3%	73.3%
	ChatGPT		80.9 ± 6.4	−11.9	−12.8%	6.8	0.82	0.88	13.3%	40.0%
	Gemini		79.9 ± 8.7	−12.9	−13.9%	7.5	0.80	0.75	20.0%	46.7%
	Foodita		82.4 ± 13.7	−10.4	−11.2%	9.2	0.76	0.64	40.0%	46.7%

## Data Availability

The data presented in this study are available on request from the corresponding author. The data are not publicly available due to institutional privacy policies regarding hospital menu databases.

## Supplementary Materials

The following supporting information can be downloaded at: https://www.mdpi.com/article/10.3390/nu18060966/s1, Table S1: Comprehensive Nutrient Estimation Accuracy Across 10 AI Models.
