# Supplementary material for: Accuracy of AI-Based Nutrient Estimation from Standardized Hospital Meal Images: A Comparison with Registered Dietitians
Source: Nutrients. 2026 Mar 18;18(6):966. doi: 10.3390/nu18060966 (PMC13029357; doi:10.3390/nu18060966)
Supplement: Supplementary file 1 [file nutrients-18-00966-s001.zip › nutrients-4158424-supplementary.pdf]

Table S1. Comprehensive Nutrient Estimation Accuracy Across 10 AI Models.

(Based on 15 Standardized Hospital Meals)

Model	NameCategory	Pearson's	rMAE (kcal)	Mean Bias (%)	Within ±10% (%)
ChatGPT-4o	LMM	0.89	38.5	-2.0%	73.3% (11/15)
Gemini 1.5 Pro	LMM	0.85	42.1	-2.9%	60.0% (9/15)
Foodita	Specialized App	0.82	48.9	-1.0%	53.3% (8/15)
Claude 3.5Sonnet	LMM	0.78	55.4	-1.2%	46.7% (7/15)
Gemini 1.5 Flash	LMM	0.746	52.1	-1.5%	40.0% (6/15)
Calomil	Specialized App	0.72	64.2	+2.5%	40.0% (6/15)
Asken	Specialized App	0.68	72.8	-3.4%	33.3% (5/15)
FiNC	Specialized App	0.55	88.6	-5.1%	20.0% (3/15)
OWN	Specialized App	0.38	128.4	+8.5%	6.7% (1/15)
CALO mama Plus	Specialized App	0.44	112.5	-7.8%	13.3% (2/15)
